# Mean Subpubic Angle of Patients Visiting Department of Radiodiagnosis of a Tertiary Care Hospital: A Descriptive Cross-sectional Study

**DOI:** 10.31729/jnma.7044

**Published:** 2022-02-28

**Authors:** Ruku Pandit, Aarati Adhikari, Hari Prasad Upadhyay

**Affiliations:** 1 Department of Anatomy, College of Medical Sciences and Teaching Hospital, Bharatpur, Chitwan, Nepal; 2 Department of Radiodiagnosis, College of Medical Sciences and Teaching Hospital, Bharatpur, Chitwan, Nepal; 3 Department of Statistics, Birendra Multiple Campus, Bharatpur, Chitwan, Nepal

**Keywords:** *gender*, *pelvic bones*, *radiology*, *sex determination by skeleton*

## Abstract

**Introduction:**

The subpubic angle in the pelvis is most often used to estimate the sex with a higher degree of accuracy. Despite the anthropological and obstetric importance of the subpubic angle, only a few studies exist in the Nepalese population. The objective of this study was to determine the mean subpubic angle of the patient visiting the department of radiodiagnosis of tertiary care hospital.

**Methods:**

This was a descriptive cross-sectional study comprising 332 pelvic digital radiographs of the patients visiting the department of radiodiagnosis of a tertiary care center from March to August, 2021. Ethical approval was taken from the Institutional Review Committee at College of Medical Sciences (Reference number: COMSTH-IRC/2021-62). Convenience sampling method was done. Demographic data like age and sex were noted. In the radiograph, the subpubic angle was measured using the program Digimizer Image Analysis Software. The collected data were analysed using the Statistical Package for the Social Sciences version 20. Point estimate at 95% Confidence Interval was calculated along with mean and standard deviation for continuous data.

**Results:**

Among 332 pelvic radiographs studied, the mean subpubic angle was found to be 120.42±22.27° (118.02-122.81 at 95% Confidence Interval). The subpubic angle in females was 137.96±12.47° and that in males was 101.10±12.56°.

**Conclusions:**

Our findings are similar to those of other studies, with a minor variation. The subpubic angle was comparatively higher in females than males in the present study. The findings of this study may be of interest to forensic scientists and obstetricians.

## INTRODUCTION

The subpubic angle (SPA) is an angle between the inferior rami of pubic bones and below the symphysis pubis.^1^ The forensic investigation of unidentified human remains begins with the identification of sex. The pelvic bones are the most precise bone for determining sex.^2^ Within the bony pelvis, SPA is one of the important parameters in sex differentiation. Sex estimate using SPA is highly accurate.^3,4^

The size of the birth canal is determined by the size of the SPA, which is a significant criterion in vaginal delivery. Determining the normal range of SPA in different populations would undoubtedly aid obstetrics in the prediction of obstructed labor and, hence, minimize maternal mortality by considering rational treatment plans. Despite the anthropological and obstetric importance of the subpubic angle, only a few studies exist in the Nepalese population.

Therefore, the study aimed to find out the mean subpubic angle in the patient visiting the department of radiodiagnosis of a tertiary care hospital of Nepal.

## METHODS

This was a descriptive cross-sectional study conducted in the Department of Anatomy of a tertiary care hospital from March to August, 2021. After obtaining clearance from the Institutional Review Committee of College of Medical Sciences and Teaching Hospital (Reference number: COMSTH-IRC/2021-62), we collected digital pelvic radiographs of patients visiting the department of radiodiagnosis. Digital radiographs of patients with hip fracture (traumatic or pathological) or misalignment at the inferior margin of the pubic bones at the pubic symphysis were excluded. Convenience sampling method was used in our study.

Sample size was calculated by the following formula:

n = Z^2^ × σ^2^ / e^2^

  = (1.96)^2^ × (16.28)^2^ / (2)^2^

  = 255

Where,

n = minimum required sample size
Z = 1.96 at 95% Confidence Interval (CI)
σ = Standard deviation calculated from maximum and minimum values
e = margin of error

However, we have included digital pelvic radiographs of 332 patients in the study. The demographic data like age and sex were also noted. All the patients were categorized into three different age groups: 18-35, 3650 and above 50.

All of the pelvic radiographs were taken according to standard anteroposterior (AP) pelvic radiograph protocols.^5^ The radiograph was taken in the anteroposterior view with the big toes contacting on their medial sides (femur in internal rotation of 10-15°) at a routine object film distance of 5cm and a focal film distance of 92cm. The SPA was measured on a digital radiograph using the program Digimizer Image Analysis Software.

For SPA, two tangent lines were drawn along the inferior border of the pubic rami. The SPA represents the angle formed by the intersection of these two lines. After the measurement in the software, the data was analyzed by using Statistical Package for the Social Science version 20. Point estimate at 95% Confidence Interval was calculated along with mean and standard deviation of SPA in males and females and in different age groups of the study participants.

## RESULTS

In this study, we examined pelvic radiographs of 332 patients, out of which the mean SPA was 120.42±22.27° (118.02-122.81 at 95% Confidence Interval), among them 158 (47.59%) were males and 174 (52.40%) were females (Table 1).

**Table 1:** Subpubic angle in malesand females (n = 332).

Sex	Maximum	Minimum	Mean±SD
Male	127.42°	70.06°	101.10±12.56°
Female	167.77°	101.01°	137.96±12.47°
Total	167.77°	70.06°	120.42±22.27°

The mean age of male patients was 44.01±17.35 years and female patients was 44.39±16.60 years while the mean age of overall patient was 44.21 ±16.94 years. The age group 18-35 had the most patients, both male and female, followed by the age groups 3650 in males and above 50 in females. The age group above 50 in males and 36-50 in females had the lowest number of patients. The SPA of three age groups were 104.40±12.48°, 101.08±12.30° and 97.59±12.56° respectively in males, while in females, the SPA was 141.08±14.60°, 138.86±9.87° and 133.78±12.47° respectively in the three age groups.

The SPA diminishes with increase in age in both sexes (Table 2).

**Table 2:** Age wise distribution of the patients and respective subpubic angle in males and females of three age groups (n= 332).

Gender	Age (years)	n (%)	Mean±SD
Male	18-35	59 (37.34)	104.40±12.48°
	36-50	44 (27.84)	101.08±12.30°
	>50	55 (34.81)	97.59±12.56°
Female	18-35	65 (37.35)	141.08±14.60°
	36-50	50 (28.73)	138.86±9.87°
	>50	59 (33.90)	133.78±12.47°

## DISCUSSION

In our study, the subpubic angle was greater in females than that in males. In both forensic and archaeological contexts, determining a biological profile from the skeleton is important. When skeletal remains are discovered, one of the first things an anthropologist looks for is the gender of an individual. The skull is the first bones of choice with the accuracy of 80-90%.^6^ If the skull is not available, the next bone considered is the pelvic bone.^7^ Sex of the pelvis can be estimated with the accuracy of 70% using single pelvic parameter whereas, combined use of multiple parameters could give the accuracy of 90% or more.^8^ The SPA is a significant criterion in sex distinction in the bony pelvis. The SPA is morphologically distinct in male and female, the shape being "U" in female and "V" in male.^8-10^ The pelvis responsiveness to sex hormones causes it to grow in the region of the ischium and pubis during puberty and adolescence, resulting in a wider pelvic outlet, an elongated pubis, and a more obtuse SPA in female,^11^ explaining the sexual dimorphism in the shape of SPA.

Traditional use of the cut-off value of 90° to differentiate the pelvis in males and females (SPA >90° indicative of female, while SPA <90° indicative of male)^3^ is widely accepted. However, several studies have shown the value of SPA coincides in males and females.^12-14^ In a present study, the SPA in male was 101.10±12.56° which is way higher than 90°. Khodeary, et al. and Kayastha P, et al. also found higher SPA (102.31±12.50° and 104.72±10.47° respectively) than the most accepted cut-off value in male.^15,16^ Mohad Ali SH, et al. discovered SPA in female was 87.4±6.5° using a threedimensional computed tomography model of the pelvis, which is less than the widely accepted cut-off value in female.^3^ Hence, the sex determination using the cut-off value of 90° is not always accurate and may not be applicable to all the population.

In our study, the SPA was 137.96±12.47° in females, whereas males had SPA of 101.10±12.56°. The findings of the present study are comparable with that of Kayastha P, et al. (SPA of male= 104.72±10.47°, SPA of female= 137.15±11.92°), who also attempted to determine the SPA using digital pelvic radiographs in the Nepalese population as in our study.^16^ On the other hand, the SPA was considerable lower in other studies; 59.05±7.11° in males and 75.60±9.81° in females in a morphometric study performed on 40 adult dry articulated pelvis^1^ and 68.6±7.6° in males and 87.4±6.5° in females in a study done in reconstructed threedimensional computed tomography pelvic model in a Malaysian population.^3^ Discrepancies in SPA made by our study in comparison with other studies may be due to variation in the modalities adopted in measuring SPA and different ethnic groups. The SPA in females was higher than in males in the current study. Similar findings were observed in several other studies.^1,15,16^

The subpubic concavity does not fully develop in females until they reach the age of 20 years.^8^ As a result, there is no sexual dimorphism in the preadolescent pelvis.^2^ Therefore, the present study was conducted on adults. The SPA diminishes with increase in age in both males and females in the current study. The distance between the ischial tuberosities of the pelvis decreases with age as the pelvis responds to the pelvis-spine balancing system.^17^ In this age-related adaptation process, the bi-tuberous diameter narrows, resulting in a reduced SPA in older people.^18^ In a similar study by Kayastha P, et al. a weak negative relationship was present between age and SPA, i.e. SPA declines as people age.^16^ Akhlaghi M, et al. also noted a significant decrease in SPA with an increase in age in females.^19^ However, Karakas HM, et al. did not find any correlation between the SPA and age,^20^ while Nwoha reported that the older individuals have lager SPA compared to the younger ones.^21^

Determination of SPA to access the sufficiency of the pelvic outlet before delivery can lower the likelihood of maternal death due to obstructed labor in the developing nations.^22^

Studies have showed variation of SPA among ethnic group.^14,19^ However, the present study did not take ethnicity into account. Hence, the result of the study cannot be applied to the overall Nepalese population.

## CONCLUSIONS

Our findings are similar to those of other studies, with a minor variation. The SPA was comparatively higher in females than males in the present study. Skeletal features reveal sexual dimorphism in populations, necessitating population-specific standards.
